# Induction Regimen in High-Risk Neuroblastoma: A Pilot Study of Highly Effective Continuous Exposure of Tumor Cells to Radio-Chemotherapy Sequence for 1 Month. The Critical Role of Iodine-131-Metaiodobenzylguanidine

**DOI:** 10.3390/cancers14205170

**Published:** 2022-10-21

**Authors:** Stefano Mastrangelo, Giorgio Attinà, Luca Zagaria, Alberto Romano, Antonio Ruggiero

**Affiliations:** 1 UOSD di Oncologia Pediatrica, Dipartimento di Scienze della Salute della Donna, del Bambino e di Sanità Pubblica, Fondazione Policlinico Universitario A. Gemelli IRCCS, Largo A. Gemelli 8, 00168 Rome, Italy; 2 Dipartimento di Scienze della Vita e Sanità Pubblica, Università Cattolica del Sacro Cuore, Largo F.sco Vito 1, 00168 Rome, Italy; 3 UOC di Medicina Nucleare, Dipartimento di Diagnostica per Immagini, Radioterapia Oncologica ed Ematologia, Fondazione Policlinico Universitario A. Gemelli IRCCS, Largo A. Gemelli 8, 00168 Rome, Italy

**Keywords:** high-risk neuroblastoma, induction treatment, chemotherapy, 131-I-metaiodobenzylguanidine (131-I-MIBG), radio-chemotherapy

## Abstract

**Simple Summary:**

Despite the use of intensive chemotherapy, the prognosis of high-risk neuroblastoma continues to be dismal. Higher cure rates appear to be mainly correlated with the response to induction therapy. Nevertheless, in recent decades, there has been no significant improvement in the response rate to most induction treatments. The present induction regimen, of only one month duration, which includes iodine-131-metaiodobenzylguanidine (i.e., low-dose rate form of irradiation that persists in the tumor cells for a few weeks) allows for a highly effective continuous exposure of tumor cells to both chemotherapy and radiotherapy, at the time of maximal tumor cell sensitivity to treatment. Following future investigations, along the same line, a higher cure rate in patients with advanced neuroblastoma might be achieved.

**Abstract:**

The prognosis of high-risk neuroblastoma (NB) continues to be poor. The early development of resistance often leads to disease recurrence. In the present study, an innovative induction regimen, including an intensive initial radio-chemotherapy sequence based on the use of iodine-131-metaiodobenzylguanidine (131-I-MIBG), was investigated. The duration of the regimen lasted only one month. Fifteen newly diagnosed patients aged >18 months with high-risk NB were treated with cisplatin, etoposide, cyclophosphamide, and vincristine, followed on day 10 by 131-I-MIBG (dose: 12–18.3 mCi/kg). Cisplatin and vincristine were administered on day 20 and 21 followed by the re-administration of vincristine, cyclophosphamide, and doxorubicin on day 29 and 30. Non-hematologic toxicity was not observed. Moderate hematologic toxicity was present probably attributable to chemotherapy. The evaluation of response was performed approximately 50 days after the initiation of treatment, yielding four complete responses, eight very good partial responses, one partial response, and two non-responses. Importantly, a complete metastatic response was achieved in 87% of patients. The present pilot study, which includes 131-I-MIBG, allows for a highly effective continuous exposure of tumor cells to both chemotherapy and radiotherapy. Furthermore, early high-dose chemotherapy followed by stem cell rescue may achieve high levels of tumor cell clearance and improve the prognosis of high-risk NB.

## 1. Introduction

Neuroblastoma (NB) is a pediatric cancer. Despite the availability of increasingly aggressive treatment, high-risk patients continue to be associated with a poor prognosis [1]. Current therapy consists of an intensive induction regimen, followed by primary tumor resection, myeloablative consolidation, and irradiation of the primary tumor site. Maintenance therapy is also employed based on both differentiation-inducing agents (e.g., retinoids) and immunotherapy. However, these treatment options have resulted in improvements only in a minority of patients [2].

Numerous patients with high-risk NB, who do not achieve complete remission (CR) after initial therapy, will die. Furthermore, the initial good response to chemotherapeutics is often followed soon by disease progression and/or metastasis. Thus, it appears that the induction regimen is the most critical step in the treatment of these patients, since resistant clones may emerge during this phase and persistence of residual resistant disease represents a real problem [3].

To avoid serious toxicity, induction chemotherapy regimens involve adequate hematologic recovery between courses. The induction schedules often last several months, while the interval between pulses of chemotherapy, with one exception (e.g., rapid COJEC) [4], is ≥3 weeks. However, it is likely that tumor cells will recover and resistant clones may develop during these intervals [3].

The main objective of the present study was to investigate an induction regimen based on an initial uninterrupted radio-chemotherapy sequence with a short duration. This regimen attempts to rapidly eradicate the tumor cells and avoid the early development of resistant clones.

NB is a radiosensitive tumor type, and external beam irradiation has been extensively used mainly for the treatment of primary tumors. Metaiodobenzylguanidine (MIBG) is a structural analog of noradrenaline; eventually, part of noradrenaline is normally recaptured by noradrenergic tissue, including NB cells; this process permits the incorporation of MIBG. Radio-iodinated MIBG is a specific and highly effective tool for the diagnosis, staging, and monitoring of NB [5]. In addition, 131-I-MIBG has the potential to deliver a very high-dose of radiation specifically to NB cells in both the primary and metastatic lesions [6,7]. Thus, it is considered one of the most active single agents in the treatment of NB. More importantly, 131-I-MIBG represents a low-dose rate form of irradiation that persists in the tumor cells for ≥2 weeks [6].

The induction regimen adopted in our center uses 131-I-MIBG and five non-cross-resistant drugs that are active against NB, administered in a rapid sequence within one month. We aimed to avoid the universally accepted chemotherapy “pulses,” with their risky intervals between courses. Therefore, the schedule allows for a continuous exposure of the tumor cells to both chemotherapy and specific high-dose irradiation [6,8].

The preliminary results of our previous study were very encouraging [6]; however, only a few evaluable patients were investigated at diagnosis. In the course of subsequent years, additional newly diagnosed patients who received the same induction regimen also showed a high rate of major and rapid responses. Moderate hematologic toxicity was the only adverse reaction observed. The extraordinary results obtained thus far in the pilot study, especially in metastatic lesions, prompted us to propose an urgently needed new therapeutic approach in an attempt to improve treatment outcomes in patients with high-risk NB.

## 2. Patients and Methods

### 2.1. Patients

The methods included in the study have been mostly described in earlier reports [6,8]. The parents of all patients who were enrolled in the study provided written informed consent for the participation of their children. There was no patient selection. The parents and referring physicians had the option to select either the experimental regimen or standard treatment. The protocol of this study was approved by the Pediatric Institutional Review Board of Università Cattolica del Sacro Cuore, Fondazione Policlinico Universitario A. Gemelli IRCCS (DIPUSVSP-25-06-2166).

In a previous study, we observed major responses in a few patients treated with high- versus low-dose 131-I-MIBG [6]. Interestingly, Matthay et al. [9] revealed that most resistant patients who received 131-I-MIBG at a dose ≥12 mCi/kg responded to treatment, whereas only one of eight patients who received 131-I-MIBG at a dose ≤9 mCi/kg achieved a significant response. Since we had experienced similar results in newly diagnosed patients after induction therapy, the cut-off of the 131-I-MIBG dose was estimated to be 12 mCi/kg.

Thus, we treated the subsequent nine patients with high-risk NB at diagnosis using a progressively increasing dose of 131-I-MIBG, starting from the aforementioned value. According to the above criteria, six additional previously investigated patients [6] were included in the present study.

Therefore, a total of 15 newly diagnosed patients with high-risk NB (INSS stage 4) were analyzed in the present pilot study; they were ordered on the basis of an increasing dose of 131-I-MIBG. Their main characteristics are shown in Table 1.

All patients exhibited good 131-I-MIBG or 123-I-MIBG uptake. There were eight males and seven females; ages ranged from 1 year and 6 months to 6 years and 9 months. Eleven patients had the primary tumor in the adrenal gland; in the remaining four patients, the NB originated in the abdomen or the posterior ganglia. All patients had distant metastases, including 14 and 13 patients with bone metastases and bone marrow (BM) disease, respectively. Patient number 9 also showed central nervous system (CNS) metastases. Of note, MYCN was amplified in five of ten patients (Table 1).

At approximately 50 days from the initiation of treatment, patients underwent disease re-evaluation using the same procedures as those used at diagnosis [6].

Response to treatment was defined according to the International Neuroblastoma Response Criteria (INRC) [10]. In addition, 131-I-MIBG or 123-I-MIBG scan SIOPEN (International Society of Pediatric Oncology European Neuroblastoma) score system was adopted [11].

### 2.2. Induction Treatment Regimen

**Figure 1:**
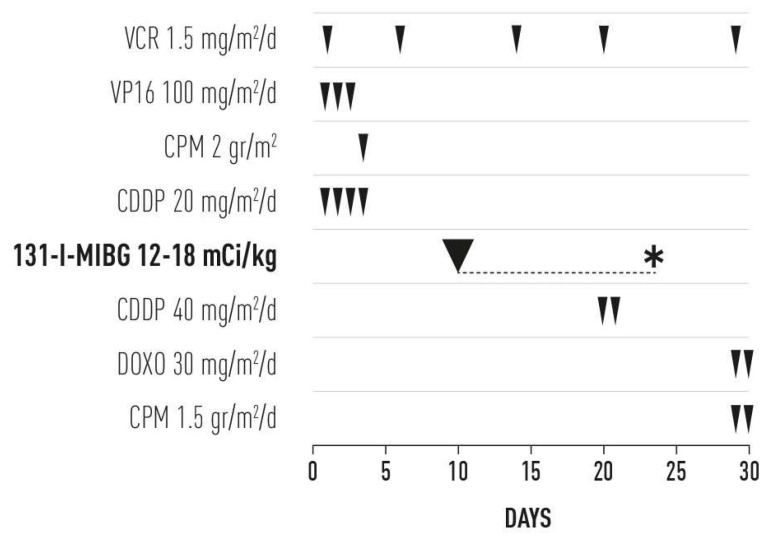
Treatment schema. VCR = vincristine, VP16 = etoposide, CPM = cyclophosphamide, CDDP = cisplatin, 131-I-MIBG = 131-I-metaiodobenzylguanidine, DOXO = doxorubicin. * Low-dose rate form of irradiation after 131-I-MIBG administration and protracted for about 2 weeks.

Within the first 10 days of treatment, we utilized four drugs which are effective against NB (Figure 1).

**Figure 2:**
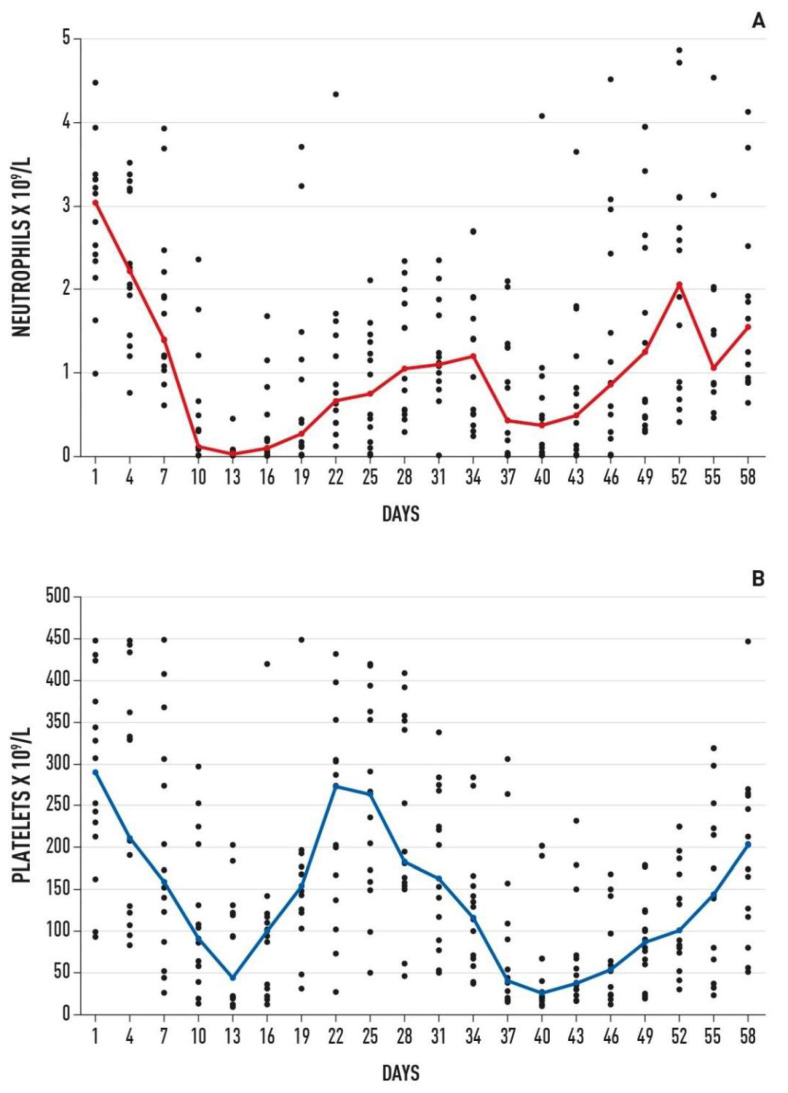
Hematologic toxicity of the 15 patients included in the study. Median and individual neutrophil (**A**) and platelet (**B**) values over time during and after treatment.

Notably, two of those drugs (i.e., etoposide and cyclophosphamide) are clearly myelotoxic. Owing to the potential risk of hematologic toxicity, to date, there are no trials which have incorporated high-dose 131-I-MIBG into an intensive chemotherapy schedule during induction therapy for newly diagnosed patients with high-risk NB. Following several attempts, our group was able to overcome this obstacle. The administration of 131-I-MIBG even at a substantial dose (≥16–18 mCi/kg) was scheduled only 10 days after initiating chemotherapy, at the time when the neutrophil and platelet counts were often very low (Figure 2).

However, there was no apparent 131-I-MIBG-induced hematologic toxicity observed. The chemotherapy–131-I-MIBG sequence was based on previous experimental work in a mouse model [12,13]. Briefly, the administration of myelosuppressive drugs to mice a few days prior to total body irradiation appeared to protect normal tissue, including BM, from radiation damage. In contrast, pre-treatment with myelosuppressive drugs did not protect tumor tissue.

As mentioned earlier in this article, 131-I-MIBG is a low-dose rate form of irradiation that persists for a few weeks after a single dose (Figure 1). Therefore, the schedule allows for a continuous exposure of tumor cells to specific high-dose irradiation, preceded and followed by chemotherapy. This continuous exposure spanned the entire initial month of treatment.

Cisplatin was included in the regimen due to its good efficacy against NB and safety profile (Figure 1). Furthermore, in vitro and in vivo preclinical studies have demonstrated synergistic effects in patients in whom cisplatin was administered a few days before irradiation or concomitantly with low-dose protracted irradiation [14].

Finally, owing to early and good hematologic recovery, further chemotherapy including two myelotoxic drugs was added to the regimen on day 29: standard-dose doxorubicin and high-dose cyclophosphamide (Figure 1). At variance with the protocol, from day 20, some patients (numbers 9, 10, 13, 14, and 15) received cisplatin (dose: 20 mg/m^2^) for 4 days. Of note, only the first 10-day treatment, which included 131-I-MIBG at the dose of 16·6 mCi/kg, was administered to patient number 12 (Table 1).

## 3. Results

### 3.1. Toxicity

Extra-hematologic and hematologic toxicities were defined according to Common Toxicity Criteria for Adverse Events version 5.0 (CTCAEv5.0) criteria.

Non-hematologic toxicity was not observed during the study period. The general condition of the patients was good, and there were only two patients that presented grade 1 vomiting and mucositis.

Figure 2 shows the time course of absolute neutrophil and platelet counts correlated with the induction regimen. The moderate hematologic toxicity observed did not cause significant infections or bleeding. Ten patients (66% of all patients) required at least one platelet transfusion for counts below 20 × 10^9^/L. Overall, approximately 10 days after initiating treatment, all patients showed grade 4 neutropenia and 10 patients developed grade 3–4 thrombocytopenia of a relatively short duration. Within 3 weeks following treatment with 131-I-MIBG, when a nadir for neutrophils and platelets may be expected, we observed hematologic recovery in most patients. In detail, 14 patients presented a neutrophil count above 1 × 10^9^/L at a median of 28 days (range 16–33), while platelet counts above 100 × 10^9^/L were reported at a median of 17 days (range 14–29) from start of treatment. Only patient number 14 showed a slow recovery of the neutrophil count, and day 29 treatment was postponed to day 45.

A similar hematologic toxicity, mitigated by granulocyte colony-stimulating factor support, was observed following the last administration of chemotherapy on day 30 (Figure 1). Thus, is it suggested that the hematologic toxicity was related to the chemotherapy, rather than 131-I-MIBG.

**Figure 3:**
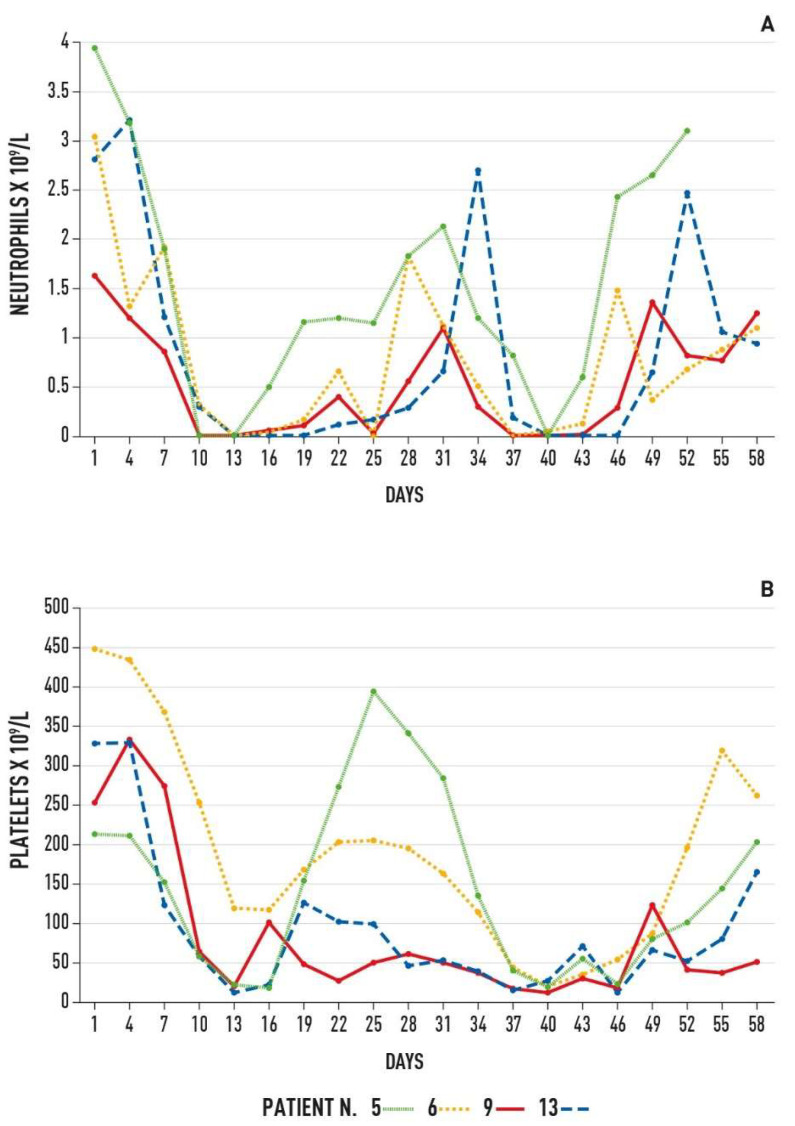
Hematologic toxicity of four patients with more extensive bone marrow infiltration. Time course of neutrophil (**A**) and platelet (**B**) values during and after treatment. Only patient number 9 was not responsive to treatment.

Finally, in contrast to the feared potential risk [15] in our patients the hematologic toxicity in the presence, at diagnosis, of extensive BM infiltration appears acceptable (Figure 3).

### 3.2. Responses

**Figure 4:**
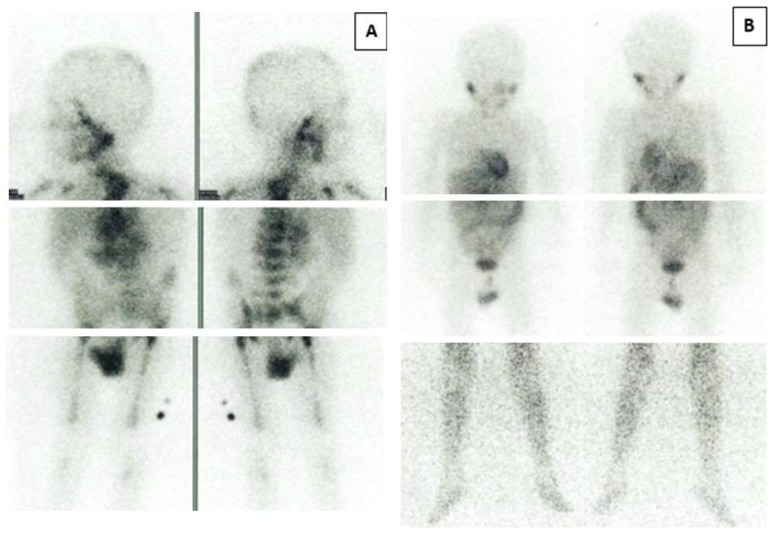
123-I-MIBG skeleton images of patient number 5. Anterior (**left**) and posterior (**right**) 123-I-MIBG skeleton images before (**A**) and after (**B**) treatment.

**Figure 5:**
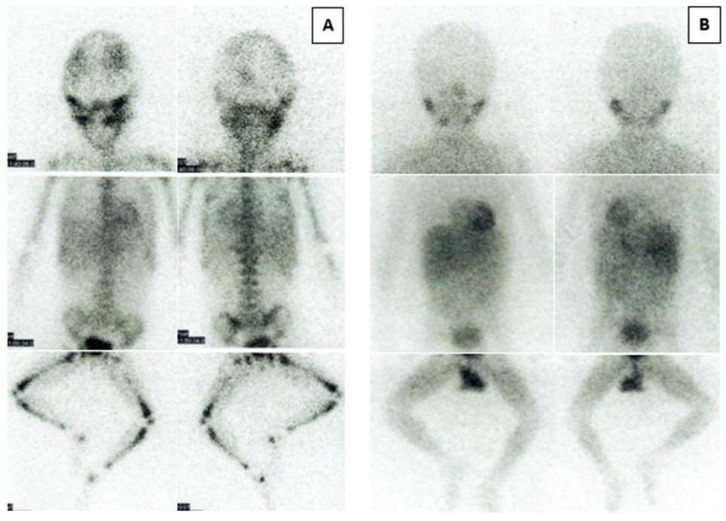
123-I-MIBG skeleton images of patient number 13. Anterior (**left**) and posterior (**right**) 123-I-MIBG skeleton images before (**A**) and after (**B**) treatment.

Table 1 shows the overall responses according to the INRC following treatment with progressively higher doses of 131-I-MIBG. The entire induction treatment lasted 30–35 days, and assessment of response was carried out at a median of 50 days (range: 43–68 days) after initiating treatment. In the 15 patients investigated we observed a marked and rapid response of the primary tumor, with more than 90% reduction of the mass in the majority of the cases (Table 1). Overall, the response rate as a sum of CR and very good partial response (VGPR) was 80% (four CR and eight VGPRs). We also observed one partial response (PR) and two non-responses. However, the most striking findings regard the complete metastatic response achieved in 87% of the patients; in particular, metastatic skeletal MIBG and BM CR rates of 85·7% and 92·3% were observed respectively. The 123-I-MIBG skeleton images of patient number 5 and number 13, with the most numerous lesions at diagnosis, are shown in Figure 4 and Figure 5. Similar results were never reported in the literature with an initial intensive treatment for only one month.

In patient number 8, with a PR, we observed a significant reduction in tumor mass (approximately 60%); however, there was no evidence of 123-I-MIBG uptake in the remaining tumor, which showed resistance to any further therapy. This observation suggests heterogeneity at diagnosis, with a dual NB cell population; this has already been described by our group [16].

Patient number 9 (one of the two non-responding patients) was treated with a course of chemotherapy (carboplatin, etoposide, and vincristine) before initiating the induction therapy protocol due to logistic problems in obtaining 131-I-MIBG on time. He had extensive CNS disease at the time of diagnosis, a type of metastatic lesion which occurs very rarely in newly diagnosed patients with NB [17]. All these patients had a particularly short survival.

On day 51, patient number 15 showed three faint skeletal 123-I-MIBG scan lesions of the original 12 lesions detected at diagnosis. These three metastatic lesions disappeared following an additional cycle of chemotherapy (carboplatin, etoposide, and vincristine) administered on day 79.

Currently, the long-term results of this innovative induction regimen are not evaluable. Despite the very encouraging results obtained, the limited number of patients analyzed and the different post-induction schedules of drugs administered preclude a concrete conclusion regarding the effect of this induction therapy regimen on outcome.

## 4. Discussion

At present, the main objective of any treatment for patients with high-risk NB should not be a prolonged survival, but an improvement in the cure rate. To reach this goal, it is essential to rapidly eradicate all malignant cells during induction therapy, in an attempt to avoid the development of resistance. In the course of time, resistant cells proliferate, expand, and are ultimately responsible for the occurrence of disease relapse and death. In most cases, the various therapeutic strategies used in induction treatment failed to prevent the development of resistant clones. Typically, more than half of patients experience disease recurrence [3].

Tumor regrowth between cycles of chemotherapy may significantly contribute to the emergence of resistant tumor cells [3]. Currently, the conventional tendency during induction treatment is a reinforcement of chemotherapy pulses. However, further dose escalation does not appear feasible because of the risk of unacceptable toxicity and the absence of a significant benefit in terms of antitumor response. Notably, even the addition of new chemotherapy courses, either before or after a standard regimen, has not significantly improved the response rate [18,19].

A potential approach to strengthening the intensity of induction treatment may be to shorten the interval between treatment courses. Toward this aim, the only attempt thus far is represented by the rapid COJEC (cisplatin, vincristine, carboplatin, etoposide, and cyclophosphamide) induction regimen. This regimen is characterized by an interval of only 10 days between chemotherapy pulses and is completed within 72 days [4]. However, to avoid the risk of hematologic toxicity, the more intensive courses (involving myelotoxic drugs) were necessarily alternated with markedly less myelotoxic (but also less effective) drug combinations.

Excellent therapeutic results were obtained in childhood leukemia [20], where a very intensive and rapid chemotherapy regimen is utilized during the first few weeks of treatment. However, the majority of effective drugs used against this disease are not myelotoxic. In contrast, most of the effective drugs against high-risk NB are myelotoxic.

The present pilot study was based on the continuous exposure of tumor cells to a short, intensive, and highly effective radio-chemotherapy sequence in newly diagnosed patients with high-risk NB. We succeeded in avoiding the potential hematologic toxicity induced by high-dose 131-I-MIBG, initially administered in a sequence with two myelosuppressive drugs (Figure 1). The hematologic toxicity, which was acceptable, appeared to be related to the chemotherapy rather than 131-I-MIBG. There was no evidence of non-hematologic toxicity in any of the patients investigated in this study.

Following treatment for only 1 month, very high percentages of CR and VGPR were observed. To our knowledge, the highly effective and rapid responses compare very favorably with past or current induction treatment results, often obtained after months of therapy [19,21].

Notably, the above mentioned rapid COJEC—one of the two most widely used induction regimens in high-risk NB—resulted in a metastatic skeletal MIBG and BM CR with rates of 35% and 72%, respectively [19]. The Memorial Sloan Kettering Cancer Center N5 induction regimen, with comparable great diffusion worldwide, resulted in metastatic skeletal MIBG and BM CR with rates of 37%, and 70%, respectively [19]. Even though in the limited number of patients included in this study, after approximately 1 month of treatment, we observed striking metastatic CR rates (i.e., 12 of 14 achieved skeleton MIBG CR, and 12 of 13 BM CR).

Our results appear related to the initial radio-chemotherapy sequence, with a major contribution by 131-I-MIBG. Interestingly, patient number 12, who only received high-dose 131-I-MIBG following the first phase of the schedule, achieved a CR. Furthermore, better responses were observed in patients treated with higher doses of the radioactive agent than in those who received lower doses (Table 1).

A wide range of 131-I-MIBG treatments have been reported in high-risk NB. These include monotherapy and 131-I-MIBG combined with chemotherapy in refractory or relapsed patients [22,23], or in combination with high-dose chemotherapy, followed by autologous stem cell transplantation [24]. Additionally, 131-I-MIBG has been used at the “front line” as monotherapy [25] and with radiosensitizers [26] with unsatisfactory responses. Recently, for the first time, the Children Oncology Group included 131-I-MIBG at a substantial dose as part of induction treatment, (at end induction) followed by a consolidation regimen. Notably, of 53 evaluable patients, 10 patients (18·9%) did not receive consolidation therapy due to progressive disease [27].

The high responses to induction treatment observed in our patients may also indicate the optimal time for the administration 131-I-MIBG in high-risk NB—a problem under investigation for several decades. Moreover, in the absence of hematologic or non-hematologic toxicity, the dose of 131-I-MIBG could be further increased. In monotherapy trials, absence of non-hematologic toxicity was observed even in patients treated with a markedly higher dose of 131-I-MIBG [28].

Both biological and clinical considerations support the rationale for the use of this innovative induction regimen.

Regarding the biological rationales, the Goldie–Coldman hypothesis suggests a decreased risk of drug resistance in tumor cells following a rapid cytoreduction in addition to the use of non-cross-resistant drugs. Such results would derive from the early combination of chemotherapy and radiotherapy. Furthermore, “chemotherapy and radiotherapy, as close together as possible, would represent the optimal treatment for a very aggressive tumor” [29], and NB is both a radiosensitive and a highly malignant tumor. Moreover, according to the Skipper–Schabel theory for advanced NB at diagnosis [30], a majority of tumor cells initially show strong chemosensitivity, causing 2–3 log cell reduction. However, resistance to drugs usually develops after the first pulses of standard-dose chemotherapy.

Regarding the clinical rationales, in a previous study [31], in high-risk NB, the evaluation of the primary tumor volume takes place at diagnosis and after 2–3 cycles of chemotherapy. Early significant sensitivity to chemotherapy has been observed even in patients with unfavorable prognostic features, MYCN amplification, or undifferentiated tumors. A similar investigation [32] showed that tumor responses occurred more rapidly during the initial two cycles, with limited change in tumor volume observed in the subsequent chemotherapy cycles. Finally, Kushner et al. [33] found that, in induction chemotherapy for high-risk NB, “early courses, which likely are most critical for achieving a significant cytoreduction”.

Our induction regimen was based on the above principles. The common denominator of both series of rationales implies that, during the early phase of induction therapy, tumor cells are very sensitive to treatment. In our opinion, it is essential to take advantage of this fact and initially utilize the most effective therapeutic agents against this tumor.

Two of our patients (numbers 6 and 9) appeared to be radio-chemoresistant ab initio. Moreover, patient number 9 presented at diagnosis with metastases in the CNS, a site associated with very poor response to treatment. The clear separation between early highly responsive and poorly responsive patients may provide an early point (within 1 month from diagnosis) for stratification by identifying a small subset of patients for whom a different treatment protocol should be implemented.

It has been shown that an early response in advanced NB during induction therapy correlates with a better prognosis. MIBG scanning, following a few courses of induction chemotherapy, showed a close correlation of early metastatic response with better prognosis [34]. Another important study concluded that a greater and early volume reduction of the primary tumor during induction therapy is associated with a better outcome [31]. Our pertinent findings during induction therapy are indeed referring to both parameters, with more intense and markedly earlier responses.

## 5. Conclusions

This pilot study showed that the present unique induction regimen (a close integration of high-dose 131-I-MIBG with intensive chemotherapy) is feasible and highly effective in newly diagnosed patients with high-risk NB. This 1-month regimen allows for the continuous exposure of tumor cells to both chemotherapy and radiotherapy. The outstanding clinical results obtained appear mainly attributed to the “optimal” use of 131-I-MIBG in advanced NB. The second important innovation of the study concerns the clear evidence of the great sensitivity of NB tumor cells to treatment during the early phase of the induction regimen. In our patients, only moderate hematologic toxicity was observed, even in patients with extensive BM infiltration. Finally, early high-dose chemotherapy followed by stem cell rescue may achieve a high level of clearance of *sensitive* tumor cells or even the eradication of tumor cells. In the literature, “megatherapy” appears to be effective only in a minority of patients, since a very drug-resistant tumor cell population prevails in most patients.

This novel induction regimen, following future investigation along the same line, may contribute to achieving a higher cure rate in patients with advanced NB.

## Figures and Tables

**Table 1:** Patient characteristics, dose of 131-I-MIBG administered, and response to treatment.

Patient Number	Age (Months)	MYCN Status	MIBG mCi/kg	Primary and Other Soft Tissue Sites	Primary Site Response	MIBG Score *		Bone Marrow #		Overall Response
						Pre-Therapy	Post-Therapy	Pre-Therapy	Post-Therapy	
1	66	amplified	12	R adr, ln	VGPR	24	0	+++	0	VGPR
2	22	unknown	12·3	R adr, ln, liv	VGPR	8	0	+	0	VGPR
3	19	amplified	13·6	abd	VGPR	3	0	0	0	VGPR
4	29	not ampl.	14·2	L adr, ln	VGPR	16	0	++	0	VGPR
5	39	not ampl.	15·6	abd, ln, thor	VGPR	27	0	+++	0	VGPR
6	81	not ampl.	15·5	ln, thor	NR	15	14	+++	0	NR
7	23	unknown	16	R adr, ln, thor, abd, liv	VGPR	11	0	+++	0	VGPR
8	53	not ampl.	16·2	L adr, ln, abd	PR	12	0	+	0	PR
9	24	amplified	16·2	L adr, CNS	NR	22	22	+++	++	NR
10	28	amplified	16·3	L adr	VGPR	6	0	0	0	VGPR
11	21	unknown	16·4	L adr	CR	19	0	+++	0	CR
12	24	unknown	16·7	L adr	CR	8	0	++	0	CR
13	27	not ampl.	16·8	L adr	VGPR	25	0	+++	0	VGPR
14	18	unknown	17	abd	CR	0	0	++	0	CR
15	55	amplified	18·3	L adr, ln, abd	CR	12	0	+++	0	CR

There is some evidence of a correlation between higher 131-I-MIBG activity and better responses. R = right, L = left, adr = adrenal gland, ln = lymphonodes, abd = abdomen, liv = liver, thor = thorax, CNS = central nervous system, CR = complete response, VGPR = very good partial response, PR = partial response, NR = no response. * SIOPEN metaiodobenzylguanidine score. # Bone marrow infiltration, evaluated by examination of bilateral posterior iliac crest aspirates and biopsy specimens, was defined as follows: +++, massive neuroblastoma infiltration; ++, moderate infiltration; +, mild infiltration; 0, no infiltration.

## Data Availability

The data presented in this study are available in this article.
